# Biosynthesized silver nanoparticles for electrochemical detection of bromocresol green in river water

**DOI:** 10.1098/rsos.221621

**Published:** 2023-08-09

**Authors:** Moustafa Zahran, Amr Mohamed Beltagi, Mahmoud Rabie, Reham Maher, Abla Ahmed Hathoot, Magdi Abdel Azzem

**Affiliations:** ^1^ Department of Chemistry, Faculty of Science, El-Menoufia University, Shibin El-Kom 32512, Egypt; ^2^ Menoufia Company for Water and Wastewater, Holding Company for Water and Wastewater, Menoufia 32514, Egypt; ^3^ Chemistry Department, Faculty of Science, Kafrelsheikh University, Kafr El-Sheikh 33516, Egypt

**Keywords:** silver nanoparticles, bromocresol green, electrochemical sensor, redox mediator, square wave voltammetry

## Abstract

In this study, silver nanoparticles (AgNPs)-based electrochemical sensor has been reported for assessing bromocresol green (BG) in river water. Firstly, AgNPs were greenly produced using the aqueous extract of *Ficus sycomorus* leaves. Then, the AgNP-modified glassy carbon (GC) electrode was prepared using the sticking method. AgNPs were characterized using transmission electron microscope (TEM), X-ray diffraction (XRD), square wave voltammetry (SWV) and scanning electron microscope (SEM). TEM and SEM were used for determining the size of AgNPs before and after adsorption, respectively. The results show that there was an increase in AgNP size from 20 to 30 nm. Additionally, XRD was used for characterizing the crystal nature of AgNPs, while SWV exhibited a characteristic oxidation peak of AgNPs at 0.06 V. Moreover, cyclic voltammetry (CV) and electrochemical impedance spectroscopy (EIS) were used for characterizing the catalytic effect of AgNPs. BG as a targeted pollutant was detected at AgNPs/GC based on its oxidation through proton and electron transfer. Two peaks corresponding to the monomer and polymer oxidation were detected. The monomer- and polymer-based sensors have revealed a linear range of 2.9 × 10^−5^ to 2.1 × 10^−4^ mole l^−1^ and low detection limits (LODs) of 1.5 × 10^−5^ and 1.3 × 10^−5^ mole l^−1^, respectively.

## 1. Introduction

Bromocresol green (BG), triphenylmethane dye, belongs to sulfone phthalein class. It has been investigated as a pH indicator and a tracking dye in DNA agarose gel electrophoresis [1,2]. Additionally, it is valuable in constructing time-temperature indicators for monitoring food quality changes. Furthermore, BG monomers can be electrochemically polymerized at the electrode surfaces, improving their electrocatalytic properties through enhanced electron transfer. For instance, poly(BG)-modified glassy carbon (GC) electrodes were previously applied for the electrochemical detection of uric acid, dopamine and ascorbic acid [3]. A poly(BG)-modified pencil graphite electrode was also employed to detect meropenem and ertapenem [4]. Additionally, the synergistic effect of poly(BG) and carbon quantum dots improved the electrocatalytic oxidation of guanine and adenine at GC [5]. Similarly, 3,4-dihydroxyphenylacetic acid and 5-hydroxyindoleacetic acid were electrochemically detected at pyrolytic graphite modified with poly(BG) and graphene nanoribbons [6]. By contrast, a carbon paste electrode based on BG and multi-walled carbon nanotube was employed to detect linezolid drugs [7].

Unfortunately, releasing organic dyes including BG dye into water causes environmental and health problems [2,8]. Therefore, treating water through the removal or degradation of BG is vital. The adsorption of BG using *Ziziphus nummularia* and chitosan poly(methacrylate) composites and its ensuing photocatalytic degradation under sunlight were reported earlier [2,9,10]. As an initial step toward the adsorption and degradation of BG, its detection techniques should be developed. Recently, spectroscopic and dispersive solid-phase extraction methods were applied for detecting BG dye [11,12]. This study reports an innovative electrochemical sensor as a simple, efficient and cost-effective tool for detecting BG.

Electrochemical sensors are considered promising tools for detecting organic pollutants with enhanced sensitivity and selectivity compared with spectroscopic and optical sensors [13,14]. Many electrochemical tools, including voltammetric, amperometric and impedimetric, could be used for pollutant detection [15–18]. The voltammetric method has been widely studied as a powerful method for dye evaluation based on different techniques, including cyclic voltammetry (CV), linear sweep voltammetry (LSV), differential pulse voltammetry (DPV) and square wave voltammetry (SWV) [19–21]. SWV is the most preferable among the voltammetric techniques because of its excellent sensitivity [22]. Therefore, this study applied the SWV technique to detect BG. For maximized sensitivity and improved detection limit, silver nanoparticles (AgNPs) alter the electrode before voltammetric determination of the targeted dye.

AgNPs have gained significant interest because of their chemical stability and other incredible physico-chemical properties [23]. Generally, their production could be accomplished through the top-down approach, which is based on turning a solid mass into smaller nanoscale particles through a mechanical method, or the bottom-up approach, which is based on manufacturing AgNPs from atomic size material through chemical processes [24]. Chemical methods depend on the direct chemical reduction of Ag+ to Ag0 through reduction agents, such as sodium citrate and sodium borohydride [25]. However, the chemical reduction methods may require further capping agents to protect AgNPs from aggregation [20]. Interestingly, biological agents, such as natural polymers and plant materials are considered valuable sources of reducing and capping agents [21,24,26–31]. Leaves, flowers and roots were studied as sources of reducing and stabilizing agents for AgNP production [29,32–34]. Numerous plant species, including *Acacia cyanophylla*, *Guettarda speciosa*, *Ficus sycomorus* and *Origanum majorana*, have been reportedly used for AgNP synthesis [24,29,35,36].

Several techniques, including ultraviolet-visible (UV-Vis) spectroscopy, fluorescence (Fl) spectroscopy, and electrochemical methods, could be employed to identify and characterize AgNPs. UV-Vis and Fl have reportedly been extensively used to confirm the presence of nanoparticles [26,28,37]. Nevertheless, few studies have reported the identification of AgNPs via electrochemical techniques, such as potentiometric and voltammetric ones [38]. A voltammetric approach is based on the oxidation of AgNPs to Ag+ with a characteristic peak. Different voltammetric techniques, such as CV, LSV, DPV and SWV, could be used to monitor the oxidation peak [31,39–41]. SWV is acknowledged for its superior sensitivity and measurement speed compared with other methods [15].

The role of *F. sycomorus* in AgNP synthesis is attributed to its flavonoid and phenolic compounds that act as reducing and capping agents [42–44]. In this study, the aqueous extract of *F. sycomorus* leaves was used for AgNP synthesis. Additionally, AgNPs were electrochemically detected using SWV. Further, the biosynthesized AgNPs have been tested as a novel electrocatalyst for the oxidation of BG for the first time. Overall, the proposed sensor is considered a valuable tool for sensitive and selective determination of BG based on greenly synthesized AgNP-modified electrode. The sensor shows high selectivity towards BG in the presence of organic and organic interferences, making the proposed sensor suitable for determining BG in real samples.

## 2. Material and methods

### 2.1. Materials and reagents

*Ficus sycomorus* leaves were sourced from Al-Shohdaa city, Menoufia governorate, Egypt. No permissions were required prior to collecting the plant leaves. The Botany Department of the Menoufia University, Egypt, recognized them. Silver nitrate as the AgNP precursor was obtained from Cambrian Chemicals. BG, methyl orange, phenolphthalein, eriochrome black T dyes and potassium ferrocyanide (k_4_[Fe(CN)_6_].3H_2_O) were obtained from Sigma-Aldrich. The components of acetate buffer solution, acetic acid and sodium acetate (CH_3_COONa.3H_2_O) were bought from Sigma-Aldrich.

### 2.2. Apparatus

Transmission electron microscopy (TEM; JEM-1230, JEOL, Japan) was used for determining the size and shape of biosynthesized AgNPs before their adsorption at electrode surface. X-ray diffraction (XRD, D2 Phaser 2nd Gen) was used also for determining the crystallinity of AgNPs. Conditions: anode = Cu; *λ* = 1.540 Å; diffraction angle (2*θ* = 10–70°). The applied voltage and the current flow were 30 kV and 10 mA, respectively. A BAS Epsilon-EC potentiostat/galvanostat was used for performing the electrochemical experiments. GC, platinum and Ag/AgCl (1 M KCl) served as the working, counter, and reference electrodes, respectively. Furthermore, a field emission scanning electron microscope (FEG-SEM; Quattro S FEG-SEM-Thermo Fisher, NL) was employed to identify AgNPs at the electrode surface. Moreover, Nanodrop spectrophotometer (Implen NanoPhotometer, N60) was used for validating real sample analysis.

### 2.3. Synthesis of silver nanoparticles

The *F. sycomorus* leaves were dried and then pulverized to a fine powder. Two grams of the powder were dissolved in 100 ml distilled water, and then the aqueous plant extract was filtered using Whatman filter paper grade No. 40. A mixture of the filtered extract (10 ml) and the aqueous solution of silver nitrate (0.002 M, 40 ml) was heated at 60°C for 20 min.

### 2.4. Preparation of silver nanoparticles/glassy carbon

First, GC was polished using alumina slurry to get a shiny mirror surface. Next, the sticking method was followed to modify GC with AgNPs, where GC was placed in AgNPs suspension (3 ml) mixed with acetate buffer solution (7 ml) for 30 min.

### 2.5. Bromocresol green detection at silver nanoparticles/glassy carbon

First, the oxidation behaviour of BG at bare and AgNP-modified GC was assessed. The bare and modified GC was dipped in acetate buffer containing BG (3.6 × 10^−5^ mole l^−1^) for 60 s before performing the electrochemical experiments. Next, BG was assessed by the modified electrode based on two oxidation peaks regarding the BG monomer and BG polymer. To optimize the detection procedure, the incubation period of the electrode in the acetate buffer containing BG dye, scan rate and pH were investigated. Different incubation times (20, 40, 60 and 80 s), scan rate values (0.02, 0.04, 0.06, 0.08 and 0.1 V s^−1^) and pH values (2.1, 3.2, 4.5, 6.4, 7.2, 8, 10 and 12) were assessed.

### 2.6. Analytical parameters

Under optimized conditions, AgNP-modified GC detects BG dye. Calibration curves were produced using OriginPro software (Origin Lab Corporation, USA). The limits of detection (LOD) and quantification (LOQ) were determined based on the calibration curves using Equations 2.1 and 2.2, respectively. The peak current was estimated based on the lines connecting the minima before and after the maxima of the peak.

2.1 $$\mathrm{Limit}\;\mathrm{of}\;\mathrm{detection}\;(\mathrm{LOD})=\frac{3\;s}{b}\;$$

and

2.2 $$\mathrm{Limit}\;\mathrm{of}\;\mathrm{quantification}\;(\mathrm{LOQ})=\frac{10s}{b},$$

where *s* and *b* are the standard deviation of the y-intercept and slope of the calibration curve, respectively. The sensor's sensitivity was measured from the slope, where sensitivity equals the slope value. Furthermore, the repeatability was determined as the relative standard deviation (RSD) from six replicates of the peak currents found by the addition of BG (3.6 × 10^−5^ mole l^−1^).

### 2.7. Interference studies

The selectivity of AgNPs/GC toward BG was investigated. The impact of methyl orange, phenolphthalein, and eriochrome black T dye as organic interferences and Na^+^, K^+^, Ca^2+^, Mg^2+^, Cl^−^, Br^−^ and I^−^ as inorganic interferences on the produced peak current was assessed.

### 2.8. Real sample studies

The validity of the proposed sensor for detecting BG in the Nile river was investigated. The water samples were obtained from the Al-Bajouria canal, Menoufia, Egypt. No permissions were required prior to obtaining water samples. The water samples were filtered, diluted twice with acetate buffer and then spiked with 1.4 × 10^−4^ mole l^−1^ BG. The concentration of BG was obtained from the calibration curve based on its SW oxidation current. The determined concentration of BG was divided by its spiked concentration and then multiplied by 100 to calculate the recovery value. Furthermore, a standard addition technique was applied to evaluate the sensor selectivity. A comparison between the usual addition and the calibration curves was made to study the effects of the possible interferences in river water.

## 3. Results

### 3.1. Synthesis and identification of silver nanoparticles

**Figure 1.**
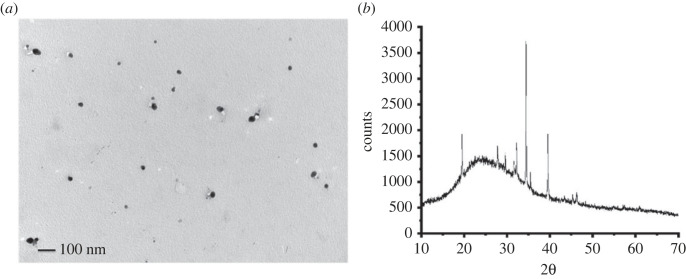
(*a*) TEM analysis and (*b*) XRD of biosynthesized AgNPs.

**Figure 2.**
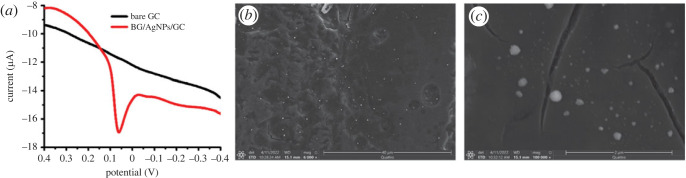
(*a*) SWV of AgNPs oxidation at GC and (*b,c*) SEM images of AgNPs/GC.

Initially, AgNPs were synthesized via heating a mixture of plant extract and AgNP precursor. The biosynthesized AgNPs are spherical with an average size of 20 nm (figure 1*a*). Then, they were electrochemically identified using SWV technique. SWV exhibited a characteristic oxidation peak of AgNPs at 0.06 V (figure 2*a*). Furthermore, the presence, shape and size of AgNPs at the electrode surface were validated using SEM (figure 2*b*,*c*). SEM images showed that AgNPs are semispherical with a diameter of 30 nm. Compared with TEM analysis (figure 1*a*), SEM images confirmed the aggregation of some AgNPs after adsorption at the electrode surface. Moreover, the XRD analysis (figure 1*b*) showed the crystallinity of AgNPs confirming the results of TEM.

### 3.2. Electrochemical characterization of silver nanoparticles/glassy carbon

**Figure 3.**
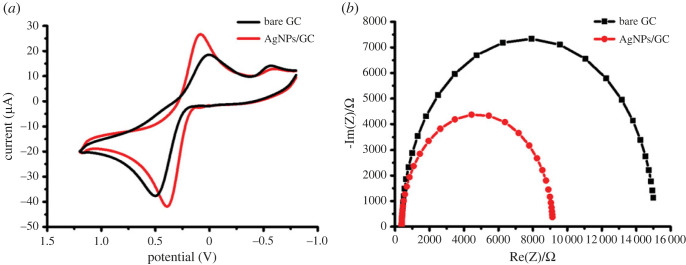
(*a*) CV and (*b*) Nyquist plot measurements performed in 0.005 M potassium ferrocyanide in presence of 1 M KCl solution.

The electrocatalytic behaviour of the bare and modified GC was assessed using CV and electrochemical impedance spectroscopy (EIS) (figure 3). CV and EIS studies were conducted in 0.005 M potassium ferrocyanide as a redox probe in 1 M KCl solution. As revealed in the CV plot (figure 3*a*), AgNP-modified GC exhibited improved oxidation and reduction peak current, including reduced peak-to-peak separation (ΔEp), compared with the bare GC showing the quick electron transfer [45]. Also, EIS is a powerful method to study the electron transfer at the electrode surface. For the EIS analysis, Randles equivalent circuit was applied where *R*_s_, *Q*, *R*_et_ and *Z*_w_ represent electrolyte solution resistance, constant phase element, electron transfer resistance, and Warburg impedance, respectively. *R*_et_, responsible for regulating the kinetics of charge transfer (one-electron transfer) at the interface of the electrode, was estimated from the diameter of the semicircle of the Nyquist plot (figure 3*b*). The value of *R*_et_ was reduced after GC modification with the biosynthesized AgNPs, verifying the improvement in electron transfer at the electrode surface [20]. Furthermore, the active surface area of the bare GC and AgNPs/GC was determined using the Randles–Sevcik equation (equation (3.1)).

3.1 $$i_{p}=(2.69\times 10^{5})n^{3/2}ACD^{1/2}\upsilon^{1/2},$$

where *i*_p_, *n*, *A*, *C*, *D* and *υ* represent current (A), the number of transferred electrons, active surface area of the electrode (cm^2^), concentration of ferrocyanide probe (mol cm^−3^), diffusion coefficient of the ferrocyanide probe (7.60 × 10^−6^ cm^2^ s^−1^) and scan rate (V s^−1^), respectively. The electroactive surface areas of the bare and modified electrodes were 0.049 and 0.082 cm^2^, respectively, which imply an increase in surface area with modification and therefore increase the active sites [21]. On the other hand, the stability of the proposed sensor has been tested. The electrochemical response of the electrochemical response has been examined during 20 days showing no change in the oxidation current confirming the stability of the sensor.

### 3.3. Bromocresol green detection at silver nanoparticles/glassy carbon

**Figure 4.**
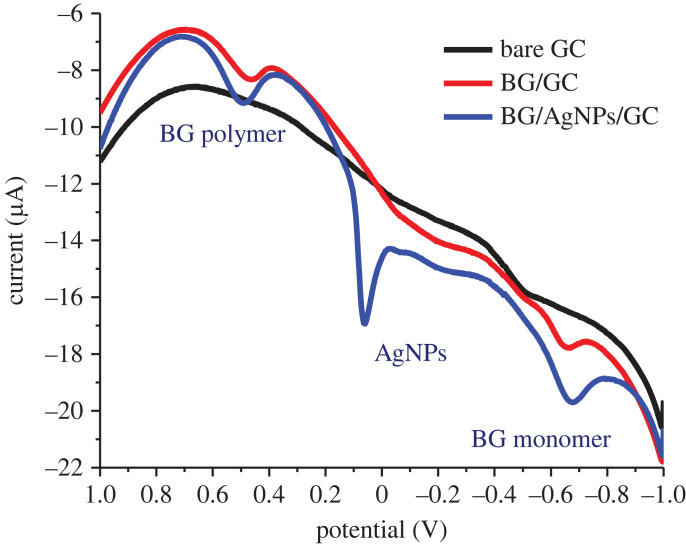
Electrochemical oxidation of BG dye (3.6 × 10^−5^ mole l^−1^) at bare and AgNPs-modified GC. Conditions: pH, 4.5; incubation time, 60 s; scan rate, 0.08 V s^−1^; step potential, 0.004 V; frequency, 20 Hz; and amplitude, 0.025 V.

**Figure 5.**
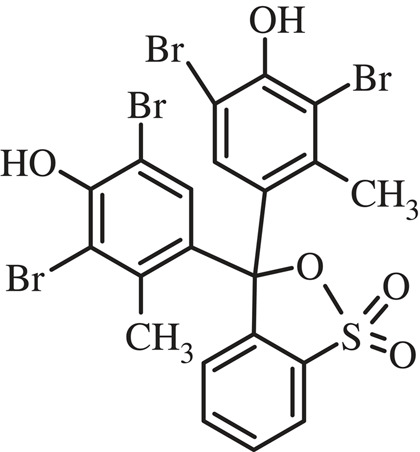
The chemical structure of BG.

**Figure 6.**
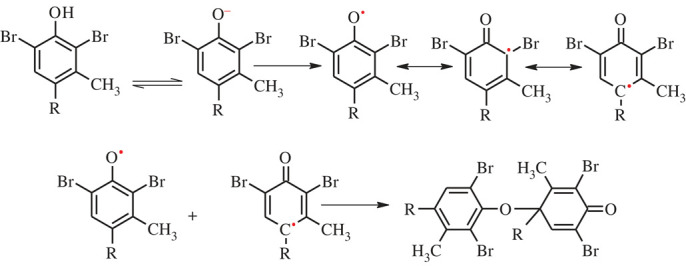
The proposed mechanism of BG oxidation and polymerization at AgNPs/GC.

**Figure 7.**
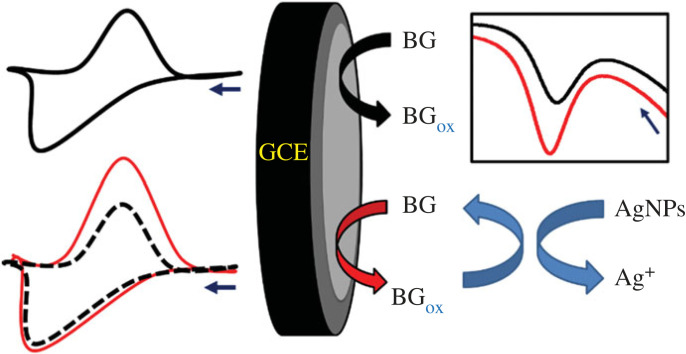
Schematic representation of redox cycling in presence of AgNPs as redox mediator.

The oxidation of BG dyes at bare and modified GC electrodes was performed using SWV. As presented in figure 4, two peaks appeared at −0.6 V and 0.5 V, possibly due to the oxidation of BG monomer and BG polymer, respectively. The chemical structure of BG and its proposed oxidation and polymerization mechanism at AgNPs/GC are presented in figures 5 and 6, respectively. AgNPs act as redox mediator for enhancing BG oxidation (figure 7).

**Figure 8.**
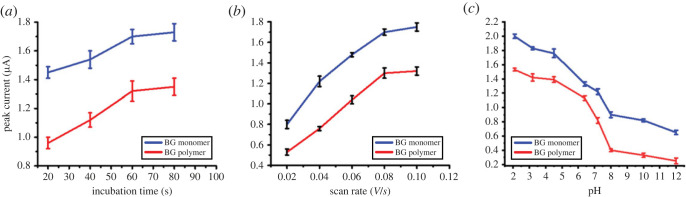
Effect of (*a*) incubation time, (*b*) scan rate and (*c*) pH on the SWV oxidation peak current of BG adsorbed at AgNP/GC. Conditions: frequency, 20 Hz; step potential, 0.004 V; amplitude, 0.025 V.

Incubation time, scan rate value, and pH were optimized to improve the BG monomer's and polymer's oxidation (figure 8). Enough incubation time is essential for efficient adsorption of BG on the AgNPs/GC before SWV analysis. Sixty seconds was an appropriate incubation period for efficient oxidation of both monomer and polymer (figure 8*a*). The scan rate is also an essential parameter for enhancing the oxidation process. A scan rate of 0.08 V s^−1^ is good for BG monomer and polymer oxidation with a high produced current (figure 8*b*). There was no change in the generated current after increasing the scan rate above 0.08 V s^−1^.

**Figure 9.**
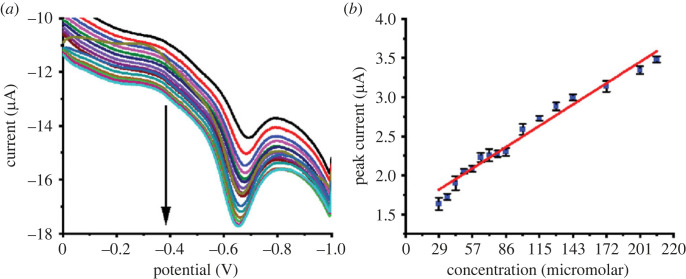
(*a*) SWV recordings and (*b*) the corresponding calibration curve (*n* = 6) for the oxidation of BG monomer at AgNPs/GC. Conditions: pH, 4.5; incubation time, 60 s; scan rate, 0.08 V s^−1^; step potential, 0.004 V; frequency, 20 Hz; amplitude, 0.025 V.

**Figure 10.**
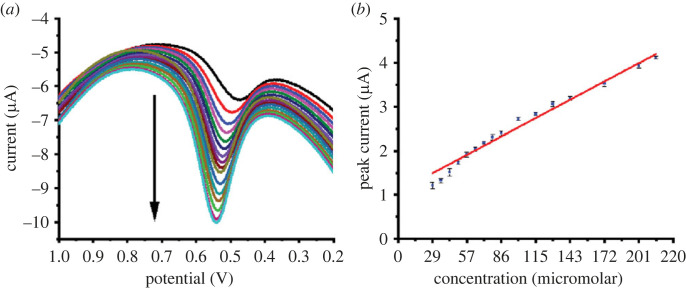
(*a*) SWV recordings and (*b*) the corresponding calibration curve (*n* = 6) for the oxidation of BG polymer at AgNPs/GC. Conditions: pH, 4.5; incubation time, 60 s; scan rate, 0.08 V s^−1^; step potential, 0.004 V; frequency, 20 Hz; amplitude, 0.025 V.

By contrast, pH has a significant impact on enhancing the oxidation process. As presented in figure 8*c*, acidic conditions in the pH range of 2–4.5 are advantageous for efficient oxidation. At strong basic conditions (pH 10–12), the efficiency of the sensor is inhibited due to the difficulty of AgNP oxidation into Ag^+^ leading to a decrease in the electron transport at the electrode surface. The decrease in electron transfer makes the dye oxidation difficult. Under optimized conditions (incubation time of 60 s, scan rate of 0.08 V s^−1^, and pH 4.5), the oxidation of different concentrations (2.9 × 10^−5^ to 2.1 × 10^−4^ mole l^−1^) of BG monomer and polymer were obtained (figures 9 and 10). The analytical parameters, LOD, LOQ, sensitivity, and RSD, were computed (table 1). Monomer- and polymer-based sensors indicated close LOD values of 1.5 × 10^−5^ and 1.3 ×10^−5^ mole l^−1^, respectively, and LOQ values of 5.1 × 10^−5^ and 4.5 × 10^−5^ mole l^−1^, respectively. Furthermore, they showed high sensitivity of 0.014 and 0.021 µA (mg l^−1^)^−1^ cm^−2^, respectively. Additionally, they exhibited accepted repeatability with RSD values of 2.12 and 1.82%, respectively. Also, the regression equations of monomer- and polymer-based sensors are I = 0.014C_BG_ + 1.54 and I = 0.02C_BG_ + 1.08, respectively.

**Table 1.** Analytical parameters.

signal	linear range (mole l^−1^)	*R*^2^	LOD (mol l^−1^)	LOQ (mol l^−1^)	sensitivity (µA (mg l^−1^)^−1^ cm^−2^)	RSD (%)
BG monomer	2.9 × 10^−5^ to 2.1 × 10^−4^	0.97	1.5 × 10^−5^	5.1 × 10^−5^	0.014	2.12
BG polymer		0.98	1.3 × 10^−5^	4.5 × 10^−5^	0.021	1.82

### 3.4. Interference study

Interferences significantly impact the validity of any proposed sensor. In this study, we studied the impact of some possible organic and inorganic interferences on the response of monomer- and polymer-based electrochemical sensors toward BG dye. The maximum acceptable concentration of each possible interference, which has led to less than 5% signal change, was determined (table 2).

**Table 2.** Maximum acceptable concentration of the possible interferences (signal change <5%).

interference	maximum acceptable concentration (mg l^−1^)^a^	
	monomer-based sensor	polymer-based sensor
Na^+^	640 ± 1.8	790 ± 1.8
K^+^	490 ± 2.2	570 ± 1.8
Ca^2+^	420 ± 1.5	520 ± 1.9
Mg^2+^	415 ± 0.9	500 ± 2.1
Cl^−^	490 ± 2.2	570 ± 1.8
Br^−^	530 ± 1.5	779 ± 1.4
I^−^	690 ± 2.3	770 ± 1.9
phenolphthalein	780 ± 1.3	850 ± 2.4
eriochrome black T	330 ± 1.7	910 ± 1.7
methyl orange	25 ± 0.8	790 ± 1.3

^a^95% confidence interval calculated (*n* = 6).

### 3.5. Real sample study

The proposed electrochemical sensor's effectiveness in assessing BG in the samples of river water was evaluated. Accepted recovery with values of 101 and 98% was reported for monomer- and polymer-based sensors, respectively (table 3). Furthermore, the standard addition technique was also followed for studying the selectivity. Regression equations of monomer- and polymer-based sensors for BG evaluation were determined as I = 0.012C_BG_ + 1.54 and I = 0.03C_BG_ + 1.08, respectively. Additionally, the spectrophotometric analysis confirmed the accuracy of the proposed electrochemical sensor (table 3).

**Table 3.** Assessment of BG in river water.

sensor	added BG	found BG	recovery (%)	spectrophotometry
monomer-based sensor	1.43 × 10^−4^ mole l^−1^	1.45 × 10^−4^ mole l^−1^	101	1.43 × 10^−4^ mole l^−1^
polymer-based sensor	1.43 × 10^−4^ mole l^−1^	1.4 × 10^−4^ mole l^−1^	98	

## 4. Discussion

In the present study, direct reduction technique was followed for the biosynthesis of AgNPs. The aqueous extract of *F. sycomorus* was used as a source of reducing and capping agents which are capable of reducing Ag^+^ to Ag^0^. Ag^0^ was then nucleated and aggregated, producing AgNPs, which were subsequently stabilized by the capping layer [20]. The AgNPs showed its characteristic oxidation potential at the GC electrode. The value of the oxidation potential is proportional to the size of AgNPs, whereas increasing the oxidation potential's value refers to AgNPs of larger size [21,31,39]. The oxidation potential of 0.6 V corresponds to an AgNP size of 25 nm [21].

The BG dye was electrochemically oxidized at the bare and modified GC electrodes. One of the hydroxyl groups of BG could be oxidized through one-proton and one-electron transfer. An improvement was observed in the oxidation peak current of the BG monomer and BG polymer following GC modification by AgNPs. This improvement in the anodic current of BG monomer and BG polymer oxidation is due to the catalytic impact of AgNPs.

Interestingly, AgNPs act as redox mediators which enhance the oxidation of BG. The SWV method is critical for achieving redox mediation or redox cycling. In each pulse, the oxidation of the BG (forward pulse) is followed by its reduction (backward pulse), and the oxidation peak current is determined from the differential current (equation 4.1) [46].

4.1 $$\Delta I=I_{1}-I_{2},$$

where Δ*I*, *I*_1_, and *I*_2_ denote the differential current, forward (direct) pulse current and backward (reverse) pulse current, respectively. In the presence of AgNPs as a redox mediator, the backward pulse current was improved, increasing the differential current (figure 7).

Generally, both monomer- and polymer-based sensors exhibited acceptable selectivity in the presence of the tested inorganic (Na^+^, K^+^, Ca^2+^, Mg^2+^, Cl^−^, Br^−^ and I^−^) and organic interferences, except methyl orange and eriochrome black T dyes, which significantly impacted the BG monomer electrochemical response. This observation is due to the similarity of oxidation behaviour of bromocresol green and the two possible interfering dyes, methyl orange and eriochrome black T dyes. Previously, methyl orange and eriochrome black T dyes were studied as redox mediators to enhance many analytes' oxidation [47–50]. Therefore, the polymer-based signal is followed instead of the monomer one in the case of such interferences.

The results of real sample study refer to the convenience of the proposed sensor for detecting BG in river water irrespective of river water interference. Additionally, there was no regarded difference between the calibration and regression equations, ensuring the effectiveness of the proposed electrochemical sensor for detecting BG in river water. For validating the proposed electrochemical sensor, the results were compared with the results obtained by spectrophotometric analysis. Spectrophotometry is a non-destructive and selective method which is used as a reliable tool for confirming the results obtained by other techniques. It was previously used for quantifying BG dye [51]. Overall, the present electrochemical sensor is convenient for the detection of BG in river water.

## 5. Conclusion

In summary, greenly produced AgNPs, which were characterized by TEM and XRD, were investigated as novel electrocatalysts for BG detection based on their direct oxidation through proton and electron transfer. The electrochemical sensor was prepared via modification of GC surface using AgNPs through the sticking method. The SWV technique and FEG-SEM confirmed the presence of AgNPs on the GC surface. Furthermore, the electrocatalytic effect of AgNPs was evaluated based on the CV and EIS experiments. Under the optimized electrolytic and voltammetric conditions, BG was detected through its oxidation at the modified GC, indicating two peaks corresponding to BG monomer and BG polymer oxidation. The analytical parameters were determined, including LOD, LOQ, sensitivity and RSD. Additionally, the impact of possible organic and inorganic interferences on the sensor electrochemical response was assessed. Furthermore, the suitability of the proposed sensor for BG detection in river water was evaluated.

## Data Availability

Source data were provided as electronic supplementary material. Source files of figures 2*a*, 3*a*, 3*b*, 4, 8*a*, 8*b*, 8*c*, 9*a*, 9*b*, 10*a* and 10*b* were submitted [52].
